# Obesity in Iran: Challenges Arising and Policy Options

**Published:** 2019-11

**Authors:** Mohamad EZATI ASAR, Mehdi HAGHI

**Affiliations:** 1. Department of Public Health, School of Health, Semnan University of Medical Sciences, Semnan, Iran; 2. Department of Health, Faculty of Health and Nutrition, Lorestan University of Medical Sciences, Khorramabad, Iran

## Dear Editor-in-Chief

Obesity is serious and most important underlying risk factor on incidence of non-communicable diseases (NCDs). Obesity in early ages has to lead to deaths caused by NCDs in production ages, consequently reduced domestic production and imposed financial burden on countries (1). In the past two decades, obesity has had an increasing trend worldwide and among all age groups. Nutrition transition is a period through which a change happens in the dietary pattern and physical activity; people's nutritional diet has become rich in saturated fats, sugar, and refined foods but poor in fiber which in turn has resulted in increase of overweight, obesity, and their related diseases (2, 3).

In Iran, the prevalence of obesity and overweight in children were respectively 12% and 13.5% (4). According to the decrease in the onset age of NCDs, this increasing trend is a warning alarm in the world and Iran. Therefore, urgent actions must be taken to prevent such diseases before outbreak of more serious problems. Malnutrition is not just the reception of insufficient food, but the other type of it is caused by receiving too much of nutrients that lead to obesity (5, 6).

Based on the conducted review studies and prevalence of obesity and overweight in adults and infants, the health system will be faced with high costs and demands for receiving services related to the obesity-relevant diseases in near future. The importance of this issue stems from the fact that incidence of NCDs can, in turn, cause irreversible effects on the health system and entire society (7, 8). Despite considerable efforts made on nutrition-related policies, nutritional document preparation, and inclusion of nutrition-related policies in national development programs, there are not special programs focusing on policies to deal with obesity have been implemented. Moreover, all stakeholders should be involved in formulation and implementation of policies to prevent from and face this phenomenon. Iran enjoys a unique healthcare network and has achieved many successes in fighting against communicable diseases (9, 10).

Now, there is a great opportunity to prevent the cost and burden of obesity-related diseases by integration of preventive programs and control of obesity in healthcare networks across the country. Due to the growing trend of obesity in Iran, the following policy options are recommended to deal with this huge monster and its complications:

Strengthening political commitment in order to ensure supplication of healthy and sustainable food; Improving technical and managerial capacities for nutrition programs in the health sector; Allocation of adequate funding to prevent and control of obesity-related diseases; Promotion of nutrition knowledge, proper attitudes, and appropriate nutrition-care methods according to the social environment and customs; Educating and empowering the health system employees about the obesity-related diseases; Identification and screening of obesity-prone groups and individuals at risk of non-communicable diseases; Evaluation of the programs and policies' consequences as well as calculation of their cost effectiveness to reduce the burden of diseases caused by obesity in different social groups; Coordination between all stakeholders who can help in prevention and reduction of obesity-related diseases; Creation of strong and consistent leadership and governance in health sector; Paying attention to at-risk-groups, especially women and children, in making policies and programming; Application of mass media to promote appropriate dietary habits; Training families through health centers to promote appropriate dietary habits; The Ministry of Education should consider and pay attention to formation of correct eating habits in school-aged children.
